# Effects of Multimodal Load on Spatial Monitoring as Revealed by ERPs

**DOI:** 10.1371/journal.pone.0136719

**Published:** 2015-09-03

**Authors:** Mario Bonato, Chiara Spironelli, Matteo Lisi, Konstantinos Priftis, Marco Zorzi

**Affiliations:** 1 Department of Experimental Psychology, Ghent University, Ghent, Belgium; 2 Department of General Psychology, University of Padova, Padova, Italy; 3 Center for Cognitive Neuroscience, University of Padova, Padova, Italy; 4 Laboratoire Psychologie de la Perception (CNRS UMR 8242), Université Paris Descartes, Paris, France; 5 IRCSS San Camillo Hospital, Lido-Venice, Italy; Wadsworth Center, UNITED STATES

## Abstract

While the role of selective attention in filtering out irrelevant information has been extensively studied, its characteristics and neural underpinnings when multiple environmental stimuli have to be processed in parallel are much less known. Building upon a dual-task paradigm that induced spatial awareness deficits for contralesional hemispace in right hemisphere-damaged patients, we investigated the electrophysiological correlates of multimodal load during spatial monitoring in healthy participants. The position of appearance of briefly presented, lateralized targets had to be reported either in isolation (single task) or together with a concurrent task, visual or auditory, which recruited additional attentional resources (dual-task). This top-down manipulation of attentional load, without any change of the sensory stimulation, modulated the amplitude of the first positive ERP response (P1) and shifted its neural generators, with a suppression of the signal in the early visual areas during both visual and auditory dual tasks. Furthermore, later N2 contralateral components elicited by left targets were particularly influenced by the concurrent visual task and were related to increased activation of the supramarginal gyrus. These results suggest that the right hemisphere is particularly affected by load manipulations, and confirm its crucial role in subtending automatic orienting of spatial attention and in monitoring both hemispaces.

## Introduction

In everyday life we are often required to perform several tasks in parallel. Under these conditions, our otherwise efficient cognitive system might reveal a number of capacity limits, sometimes fairly severe [1]. The limitations of our spatial abilities under attentional load have a number of practical consequences. For example, activities requiring complex spatial processing, such as driving, are implemented with more effort when we perform another task in parallel, such as talking to a person [2]. It has been known since the 1960s that performing an auditory task or increasing visual demands at fixation makes detection of peripheral visual targets slower and less accurate [3], giving rise to what has been called “tunnel vision” [4]. More recently, the studies addressing this fascinating issue, namely the effects of attentional demands on visual perception, have been mostly framed within the framework provided by Lavie’s [5,6] influential “load theory” of attention. The load theory focuses on the processing of visual information. It provides a comprehensive explanation of how the influence of peripheral distractors depends on the ‘load’ of a current attentional task at fixation, with less interference occurring when more attentional capacity is demanded by the processing of the central stimuli. Specifically, higher load for central position would lead to the exclusion of irrelevant peripheral inputs at an earlier stage of visual processing, as compared with lower central load. A less common approach to investigate the effect of task load in healthy participants consists in using task-relevant, lateralized targets. A similar context is ubiquitous in everyday life behaviour (e.g., talking on the phone while driving) and it is also particularly relevant for a leading theoretical proposal regarding the functional architecture of attention networks in the brain [7]. Although (or because) several alternative terminologies exist, a neutral taxonomy of the manipulations typically performed in load studies distinguishes top-down from bottom-up approaches. Top-down manipulations consist of a change in the instructions (respond to a given color only vs. respond to a given color and a given shape) while stimuli are kept constant. In contrast, in bottom-up manipulations different levels of attentional load are obtained with a physical change in the stimuli, whereby instructions (tasks) are kept constant.

While not much is known about the interplay between spatial and non-spatial attention in the intact brain, some important hints about the effects of load are provided by studies requiring brain damaged patients to implement overt responses to lateralized targets. Indeed, a wealth of neuropsychological findings converge in showing that increased load results in asymmetrical processing of peripheral targets after unilateral brain damage. There is now consensus that right-hemisphere-damaged patients show more severe rightward bias/leftward omissions whenever a concurrent task is performed. This disruption occurs regardless of the nature of the concurrent task, that is, whether visual [8–13] or auditory [14] or both visual and auditory [15,16]. Patients data thus suggest that the right hemisphere, known to be prominently involved in spatial monitoring, is also particularly sensitive to the general increase in attentional load. These recent findings extend the fact that the mere disengaging from a central position poses a challenge to right brain-damaged patients [17,18]. Attentional asymmetries can be easily explained by the selective impairments of bottom-up mechanisms characterizing neglect [19].

We recently implemented a novel, exclusively top-down, manipulation of attentional load in a series of clinical studies on right-hemisphere damaged patients [15,16]. Our experimental paradigm used peripheral stimuli as task-relevant targets and load was increased through the use of multi-tasking. That is, one additional stimulus, either visual (at fixation) or auditory (binaural) had to be concurrently reported in the dual-task condition. Chronic right-hemisphere damaged patients showed striking awareness deficits for contralesional hemispace when performing dual-tasks [20], whereas their spatial monitoring performance was relatively spared in the single task condition. A dramatic drop in performance under dual task occurred not only for bilateral targets (i.e., indexing extinction [15,16]) but also for single, unilateral, contralesional stimuli (i.e., hemispatial neglect [21]). The load manipulation effectively resulted in a pattern of omissions for left-sided targets also in several patients who did not show neglect according to standard, paper-and-pencil, tests [21]. The load-induced deficit selectively affected the contralesional side, and was not present in matched controls [15,16]. Intriguingly, at the group level, the effect of dual-tasking was identical across the two conditions (visual vs. auditory [21]). This suggests that performance in spatial monitoring was hindered by the recruitment of unspecific, amodal, attentional resources, rather than to modality-specific (e.g., visuospatial) load. Whether the latter finding has a counterpart in normal brain functioning is an issue that remains to be investigated. Several fMRI studies investigated the neural correlates of spatial processing under attentional load [13, 22–27]. It has been shown [23] that increased visual load strongly modulates neural activity in the primary visual cortex (V1) of healthy participants, also in the absence of conscious perception. Moreover, a rather consistent finding when visual working memory is loaded is the suppression of activity in temporoparietal areas [22, 25, 27], which occurs at early stages during working memory encoding [22]. An fMRI study in right parietal stroke patients [13] showed reduced activation in right primary visual areas (left hemispace) following increased attentional load at fixation, thereby calling for the presence of an early-gating neural mechanism.

Electrophysiological studies investigating how and when attentional load modulates ERPs have provided a complex picture and somehow contradictory findings, probably due to the close dependence of early components on the specific characteristics of the manipulation performed. For instance, it has been shown that increased task load can produce opposite effects depending on whether the peripheral stimuli are relevant or irrelevant for the task. More specifically, increased amplitudes were observed for peripheral stimuli presented as targets (i.e., task-relevant), whereas decreased amplitudes were observed for peripheral stimuli presented as distracters (i.e., task-irrelevant) [28]. The timing of the effect of load, that is, at which processing stage it can modulate the ERPs, is a hotly debated issue. Studies using task-irrelevant peripheral distracters have reported that the amplitude of the early positive component appearing around 100 ms after stimulus presentation (P1) decreases with increasing attentional load at fixation [29]. Using multiple, peripheral, task irrelevant distracters, some studies have shown that attentional load can even influence the first sweep of visual processing, as revealed by the C1 component [30–32]. However, also contrasting results have been reported [33–35]. Some authors have concluded [35] that attentional load does not exert its influence on P1 but only at later stages.

Load effects have been more consistently described for the N1 (around 200 ms post-stimulus) and for later components/higher level processes [36,37], which are known to depend on attentional deployment [38,39]. It is worth noting that most, if not all, the studies focusing on the effects of non-spatial load manipulations adopted stimuli that were visual in nature and therefore spatially characterized [36,38]. To the best of our knowledge, all previous studies that manipulated spatial monitoring difficulty through the addition of a concurrent task with a different nature focused on the factors affecting multisensory integration [40].

A recent ERP investigation with healthy participants addressed the role of visual load at fixation (rapid serial visual presentation of a stream of alphanumeric stimuli) on the detection of peripheral targets [41]. Participants performed either a single-feature (low load) or a conjunction search (high load; further details in [41]) and they also responded to peripheral targets presented for 400 ms. The typical N1 enhancement for contralateral visual stimulation [42] was reduced under high load over the occipital and the inferior parietal regions of the right hemisphere. Another ERP study on load effect in healthy participants focused on the interaction between task load and spatial attention [36]. It showed the modulation of N1 component in an oddball task in which attentional selection was performed on the basis of spatial or color features of stimuli [36]. Visual load level was bottom-up manipulated by varying the similarity between a standard and a target stimulus, whereas type of load (always in the visual modality) was manipulated by changes in the feature that had to be processed (either color or position). Load increase similarly affected the difference waveforms between attended and unattended stimulus dimensions in both position-based and color-based tasks.

In the present study, we exploited the sensitivity of ERPs to investigate how the neural dynamics of visuospatial monitoring are influenced by concurrent task demands. The unparalleled temporal resolution offered by ERPs is particularly valuable when the time-course of the neural effects of top-down attention needs to be established [43]. Moreover, the study of ERPs is particularly appropriate for manipulations implying a constant sensory stimulation and only a change in task instructions (The Hillyard Principle, see [44] p. 68). Indeed, we investigated for the first time the effect of top-down, multi-modal manipulations of attentional load in healthy participants using the same type of experimental paradigm that induced severe contralesional awareness deficits in right brain damaged patients [15]. The fact that our paradigm requires an overt response to the lateralized stimuli constitutes a first important difference with respect to most previous studies on the neural correlates of attentional load [13, 26, 30, 32], which might be important for predicting the impact of attentional load in settings where peripheral stimuli may have ecological relevance. A second important characteristic of our method is that the dual-tasking procedure is implemented by asking participants not only to monitor one additional aspect of the stimuli but also to provide a second response. A third crucial feature is that it includes a concurrent task that is neither spatial nor visual in nature, thereby assessing the effect of cross-modal load on visuospatial processing.

In summary, we aimed to determine when and how an increase in attentional load through a dual-task manipulation modulates electrophysiological markers of visual processing of task-relevant stimuli and to what extent the effect of load is modality-specific (visual vs. auditory). Based on the findings of O’Connell et al. (2011), who implemented a detection task while load was manipulated within the visual modality, we expected a significant reduction of the contralateral N1 amplitude under load. We predicted similar effects of visual and auditory load on the basis of our previous clinical finding: in the presence of brain damage spatial awareness was modulated by dual-tasking regardless of the nature (i.e., sensory modality) of the stimuli that had to be concurrently processed [19]. While we had no a-priori hypothesis regarding earlier P1 and late N2 components, the present paradigm offers a new way to address the issue of whether these components are modulated (or not modulated, [35,41]) by attentional load.

## Materials and Methods

### Participants

Fifteen undergraduates (8 males; mean age: 22.3 years) were tested at the Department of General Psychology of the University of Padova. All participants were right-handed (average score > 80%, according to the Edinburgh Handedness Inventory [45]) and had normal or corrected-to-normal vision. All participants were more than eighteen years old and gave their written informed consent to take part in the experiment, according to the Declaration of Helsinki. The experimental procedure was approved by the Ethics Committee of the Department of General Psychology, University of Padova.

### Stimuli, tasks, and procedure

Participants sat at a distance of about 60 cm from a 38 x 30.5 cm computer monitor. The task was programmed and administered using E-Prime (Psychology Software Tools, Pennsylvania, USA, http://www.pstnet.com).

**Fig 1:**
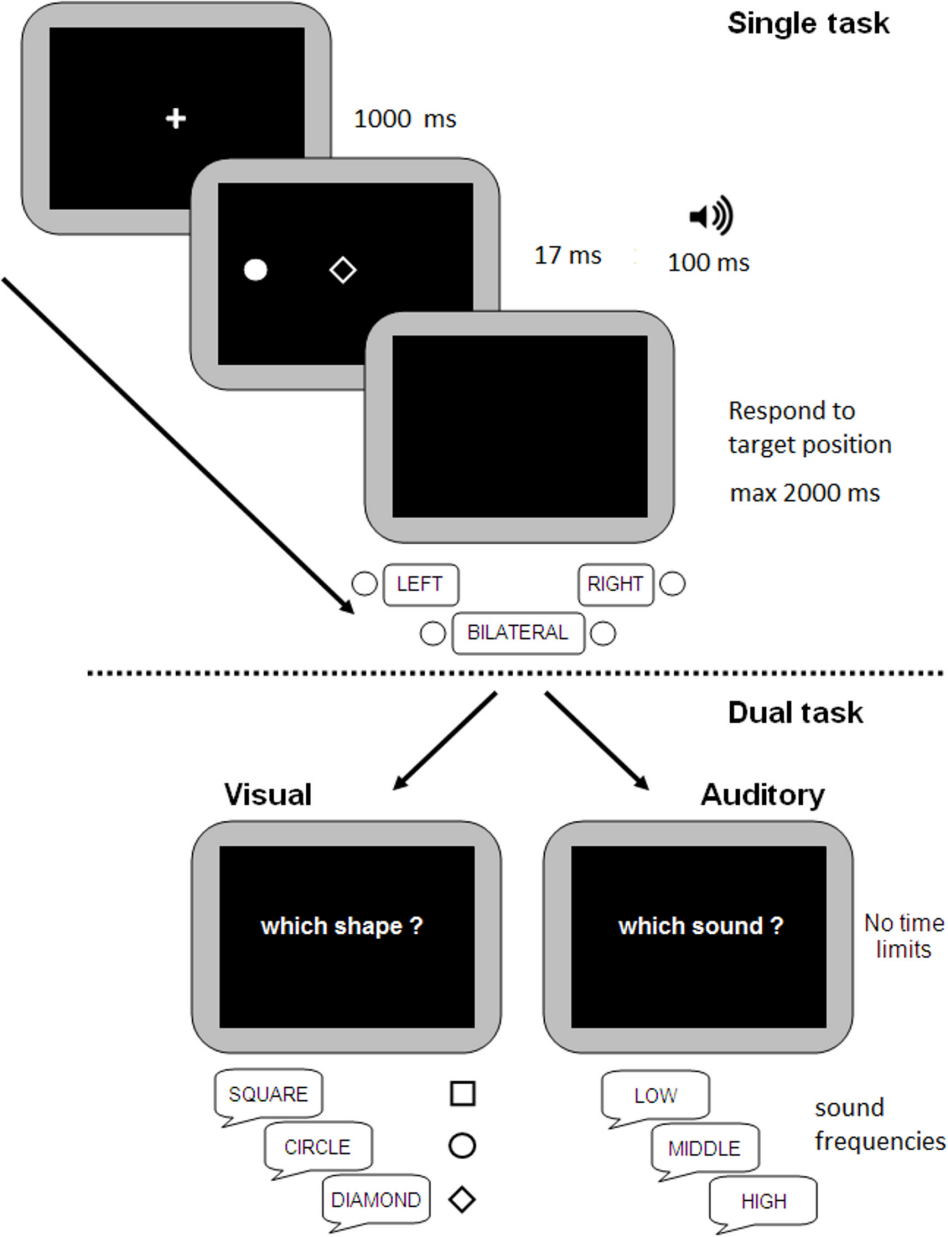
Trial structure of the Single task (top panel) and of Dual tasks (bottom panel). Across all tasks several stimuli were presented: lateralized dot(s), a central form and a binaurally presented sound. In the Single task participants only had to report the position of the dot. In Dual tasks after the response to dot(s) position, participants had to report the identity of the central shape (Visual Dual-task: left side, bottom panel) or the pitch of the sound (Auditory Dual-task: right side, bottom panel).

There were three experimental tasks (Fig 1): one single-task condition and two dual-task conditions (visual vs. auditory). Each trial started with a black screen (1000 ms). A black background was present through the whole experiment. Then, a white fixation cross was centrally-presented for 1000 ms. A white dot target (approximately 0.8° of visual angle) was then presented, in equal proportion, on the left side, on the right side, or bilaterally for 17 ms, at a lateral distance of about 16° of visual angle from the centre of the screen. Therefore, either a single target (left-sided or right-sided) or bilateral targets (left- and right-sided) were presented. Synchronously with the lateral target(s), a geometric shape (square, circle, or diamond, in equal proportion, about 1.1° of visual angle) was presented at fixation and a pure tone (high frequency ≈ 800 Hz, medium ≈ 450 Hz, or low ≈ 255 Hz, in equal proportion) was binaurally presented by means of earphones. After the offset of sound (100 ms), a blank screen was presented. Then, different characteristics of stimuli had to be reported according to the to-be-performed task.

In the Single task, participants had to report the position of the target(s) (i.e., “right”, “left”, or “both” sides), while ignoring the central shape and the auditory tone. Participants were required to respond as fast and as accurately as possible by means of three response keys (left index for left target, right index for right target). For bilateral targets, either a response with left or right thumb (counterbalanced between participants) was requested. Absence of response at the end of the trial (2 sec) was considered an omission. In both Dual tasks, the display and the sequence of events were identical to that of the Single task. In the Visual Dual task, participants had to respond (using the keyboard) to the position of the lateral visual target(s) and then verbally classify the centrally presented shape. In the Auditory Dual task, participants had to respond (keyboard) to the position of the lateral visual target(s) and then to verbally classify the sound pitch as high, medium or low. The experimenter coded participants’ oral responses to the identity of the centrally presented shape (in the Visual Dual task) or to the sound pitch (in the Auditory Dual task).

Each task comprised 108 trials, equally distributed in two blocks (2 repetitions x 3 sounds x 3 shapes x 3 spatial positions), for each task. Participants performed the Single task first, and then the Dual tasks (Visual vs. Auditory) in a counterbalanced order. We decided to avoid full counterbalancing because it could have resulted in potential carry-over effects. Indeed, when presenting the single task after the dual task(s) participants would have had to ignore both concurrent stimuli (form and sound) shortly after they were task-relevant in the dual-task(s). The importance of maintaining gaze at fixation was stressed before each block.

### Data acquisition and analysis

EEG cortical activity was recorded by 32 tin electrodes, 30 mounted on an elastic cap (ElectroCap) according to the International 10–20 system [46], and the other two applied on mastoids (M1, M2). Eye movements were recorded from two additional electrodes placed below the right eye (Io1) and on the left canthium (F9), respectively. The electro-oculogram (EOG) was therefore recorded with a bipolar montage. All cortical sites were on-line referred to M1. Data were stored using the Micromed software (System Plus, Micromed, Mogliano Veneto, Italy). Data were recorded with a 0.2–30 Hz bandwidth; the sampling rate was set at 512 Hz and the impedance was kept below 5 kΩ.

EEG was continuously recorded in the AC mode and stored for later analysis. Data were off-line re-referenced to the average reference (including the activity of both mastoids). Signal analyses were carried out using the Brain Vision Analyzer system (Brain Products GmbH, Germany). Eye movement artifact components (i.e., vertical and horizontal movements, and blinking) were corrected by applying the Independent Component Analysis (ICA) transformation to the EEG signal. Raw data were therefore segmented in epochs of 1.5-s intervals, including 0.5 s before and 1 s after target onset, and a 100-ms baseline preceding target onset was subtracted from the whole trial epoch. Each trial was then visually inspected for any residual artifacts (e.g., head movements or muscular activity), and trial corresponding to wrong responses to either target position, form/sound type or both, were discarded. All accepted trials within a specific experimental condition (on average, 63.1%, with no differences between conditions) were averaged. Thus, this rate of averaged epochs included all artifact-free trials for which participants provided correct responses to both the target and, for Dual tasks only, the secondary task (shape or sound classification).

**Fig 2:**
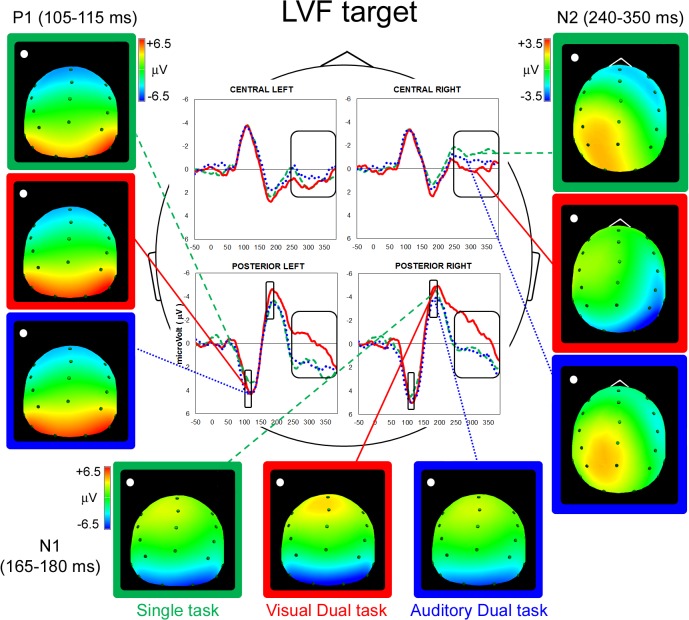
Left Visual Field: Grand mean average potential and spline maps. P1 (left side), N1 (horizontal) and N2 (right side) components of LVF stimuli during Single (dashed green lines), Visual Dual (full red lines) and Auditory Dual task (dotted blue lines) are represented. Negativity is shown upwards.

**Fig 3:**
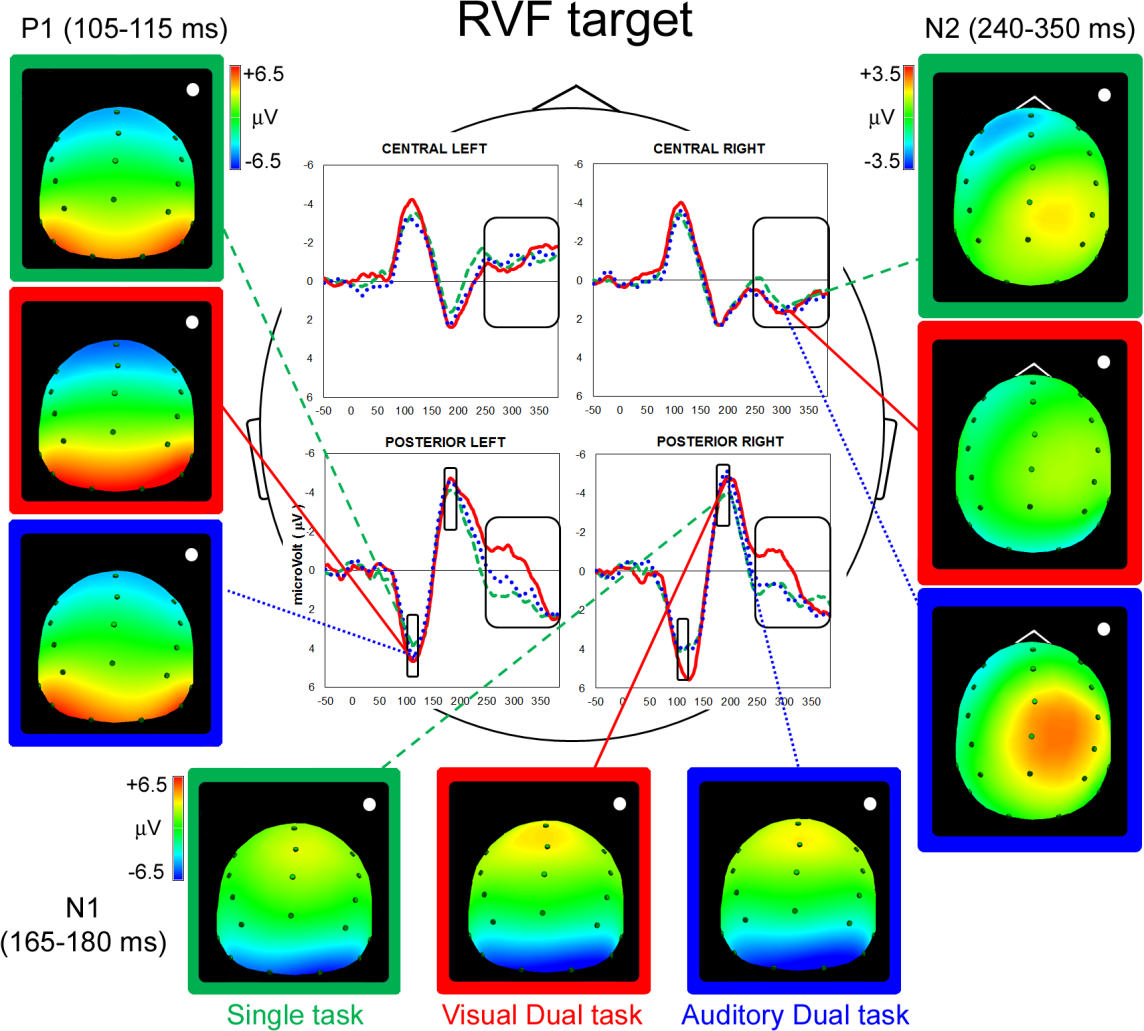
Right Visual Field: Grand mean average potential and spline maps. P1 (left side), N1 (horizontal) and N2 (right side) components of RVF stimuli during Single (dashed green lines), Visual Dual (full red lines) and Auditory Dual task (dotted blue lines) are represented. Negativity is shown upwards.

On the basis of grand-mean waveforms (Figs 2 & 3), we analyzed the time-windows centered on P1, N1 and N2 peaks (i.e., 105–115 ms, 165–185 ms and 240–350 ms, respectively). The Kolmogorov-Smirnov test was applied to ensure that every ERP component was normally distributed (all ds ≤ 0.23642, ps > 0.20). For statistical analysis, electrodes were clustered into four quadrants/regions of interest: Central Left (CL: F3, FC3, C3), Central Right (CR: F4, FC4, C4), Posterior Left (PL: P3, P7, O1) and Posterior Right (PR: P4, P8, O2). The mean amplitude values of the potentials measured in sites of the same polarity were used: thus, for P1 and N1 components only posterior electrodes were considered, whereas for the late N2 components separate analyses have been carried out on central and posterior sites.

Since the responses to bilateral targets were executed by half of the participants with the left and by half of the participants with the right thumb, the analyses of the late event-related component (i.e., the N2) would require to consider separately these two subgroups, at least on central sites corresponding to response-related contralateral negativity. This would yield an insufficient number of participants for performing reliable statistical analysis. We therefore deemed it as more appropriate to focus only on the ERP components evoked by unilateral (i.e., left-sided or right-sided) dots, for which data from all participants were available, on posterior regions and, limited to late N2 component, on central sites.

In addition to standard behavioral and ERP analysis we used sLORETA/eLORETA (standardized/exact Low Resolution Electromagnetic Tomography; [47]) to compute the smoothest possible 3D-distributed current source density solution constrained to grey matter. This approach was particularly suited for our analysis given that, due to the smoothness constraint, it does not need an a priori number of known sources. Finally, to focus specifically on load effects, we performed, separately for each component, a cost analysis by subtracting the activation found in the single-task from the activation found under dual-tasks.

## Results

Both behavioral and ERP data were analyzed using repeated measures analysis of variance (ANOVA). The Greenhouse-Geisser (GG) correction was applied in the case of violation of sphericity (in these cases, we report uncorrected degrees of freedom, epsilon values, and corrected probability levels). Post-hoc comparisons were computed using the Newman-Keuls test (p < 0.05). All analyses have been carried out using the Statistica software (Statsoft Italy, 6.1 version). Only significant main effects or interactions are reported.

### Behavioral Results

Reaction times were analyzed by means of a two-way ANOVA with Task (three levels: Single vs. Visual Dual vs. Auditory Dual) and Target Position (two levels: Left Visual Field [LVF] vs. Right Visual Field [RVF]) as within subjects factors. RTs (Table 1) were faster in the Single task (415.7 ms) compared with those measured in both Dual tasks (Visual: 481.8 ms, Auditory: 462.2 ms; all ps < 0.01; Task main effect: F(2,28) = 12.13, p < 0.001, GG ε = 0.79). Cohen’s d values (from now on reported as "d") were 1.03 and 0.73, respectively. No other effect was significant (Target position: F(1,14) = 1.78, p = 0.20).

**Table 1:** Behavioral data. Mean and Standard Deviations (SD) of Response Times (RTs) and Accuracy as a function of Target position and load condition.

Target position	Task	RTs	Accuracy
		(ms ± SD)	(% ± SD)
**LVF**	**Single**	**412 (± 31)**	**98.1 (± 2.5)**
	**Visual Dual**	**471 (± 74)**	**98.6 (± 1.9)**
	**Auditory Dual**	**452 (± 74)**	**98.3 (± 3.6)**
**RVF**	**Single**	**420 (± 45)**	**98.4 (± 2.2)**
	**Visual Dual**	**492 (± 97)**	**98.1 (± 2.8)**
	**Auditory Dual**	**472 (± 96)**	**99.4 (± 1.2)**

Accuracy data were analyzed by fitting a generalized-linear mixed effects model with the logit as link function, using R [48] and the *lme4* library [49]. The model had Task and Target Position as within subject predictors; statistical significance was assessed using likelihood ratio tests. This analysis did not reveal any significant effect (interaction between Task and Target Position: χ^2^(2) = 3.43, p = 0.18; Task main effect χ^2^(2) = 1.41, p = 0.49; Target Position main effect χ^2^(2) = 0.55, p = 0.46).

### ERP results

We performed separate three-way ANOVAs on P1, N1 and N2 time-intervals with the following factors: Task (three levels: Single vs. Dual Visual vs. Dual Auditory), Target position (two levels: LVF vs. RVF) and Laterality (two levels: Left vs. Right hemisphere).

#### P1 component (posterior sites)

**Fig 4:**
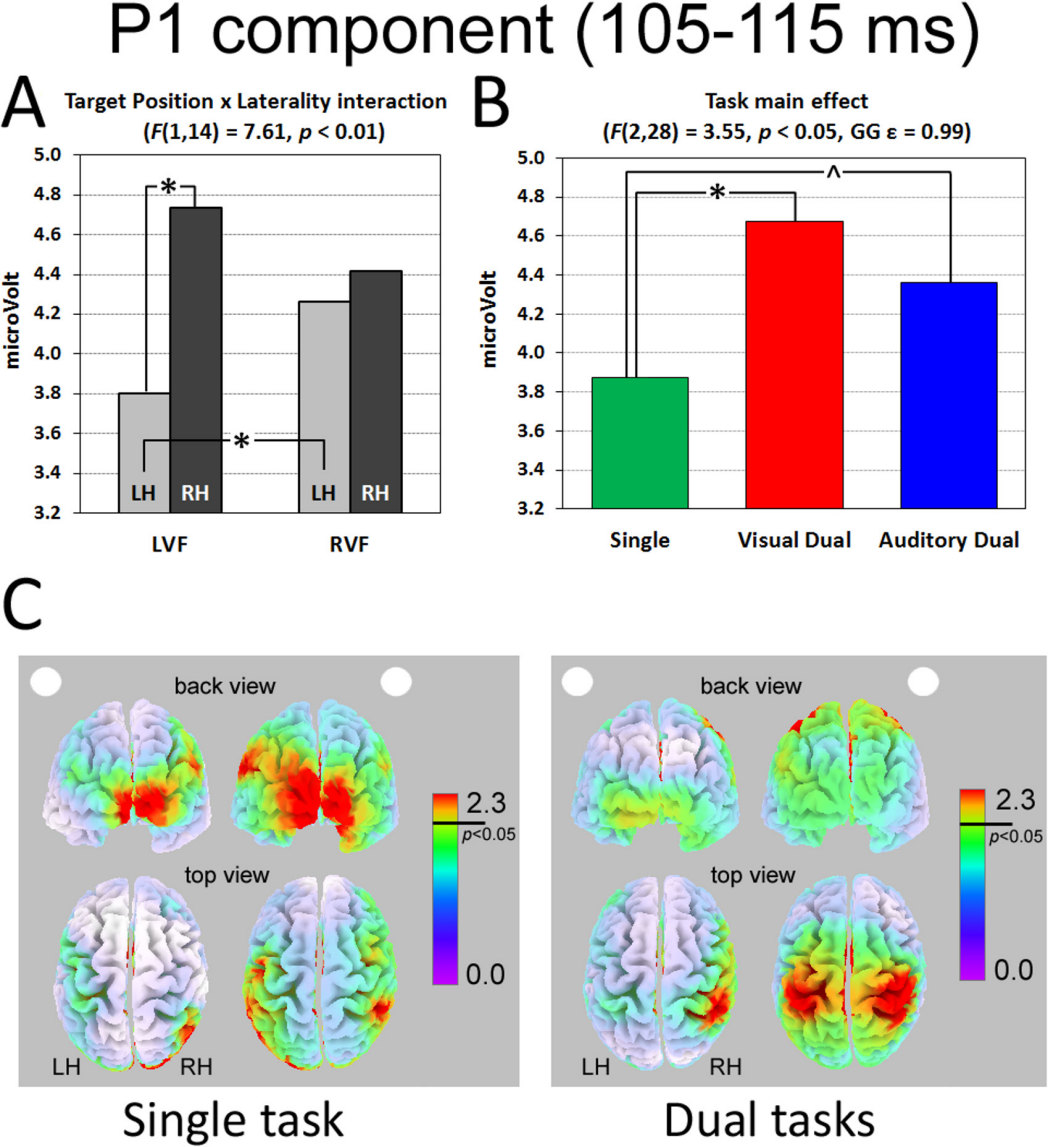
P1 component elicited on posterior sites. (A) Target Position by Laterality interaction, (B) Task main effect and (C) sLORETA source analyses of LVF and RVF stimuli during Single (left panel) and both Dual tasks collapsed (right panel). The white dot (on the left or on the right) indexes target position. * significant post-hoc comparisons. ^ *p* = 0.07. LH = Left Hemisphere; RH = Right Hemisphere.

The ANOVA carried out in the early time interval corresponding to the P1 component (105–115 ms after target onset) revealed a significant interaction between Target Position and Laterality (F(1,14) = 7.61, p < 0.01). LVF targets elicited larger right than left positivity (p < 0.01, d = 0.48), whereas RVF targets evoked similar, bilateral, activation (d = 0.08; Fig 4A). When considering within-hemispheres differences, greater positivity was measured, in the left hemisphere only, for RVF than for LVF targets (p < 0.05, d = 0.27; Fig 4A).

In addition, the significant main effect of Task (F(2,28) = 3.55, p < 0.01, GG ε = 0.99) indexed greater positivity under dual tasking (Visual Dual task: 4.67 μV, d = 0.42; Auditory Dual task: 4.36 μV, d = 0.24) than in the Single task (3.87 μV, p < 0.05 and p = 0.07, respectively; Fig 4B). It should be noted that this increased amplitude under load occurred concurrently with a posterior-to-anterior shift of the component generator (see Fig 4C and the Source Analysis below). No other main effect or interaction was significant.

**Fig 5:**
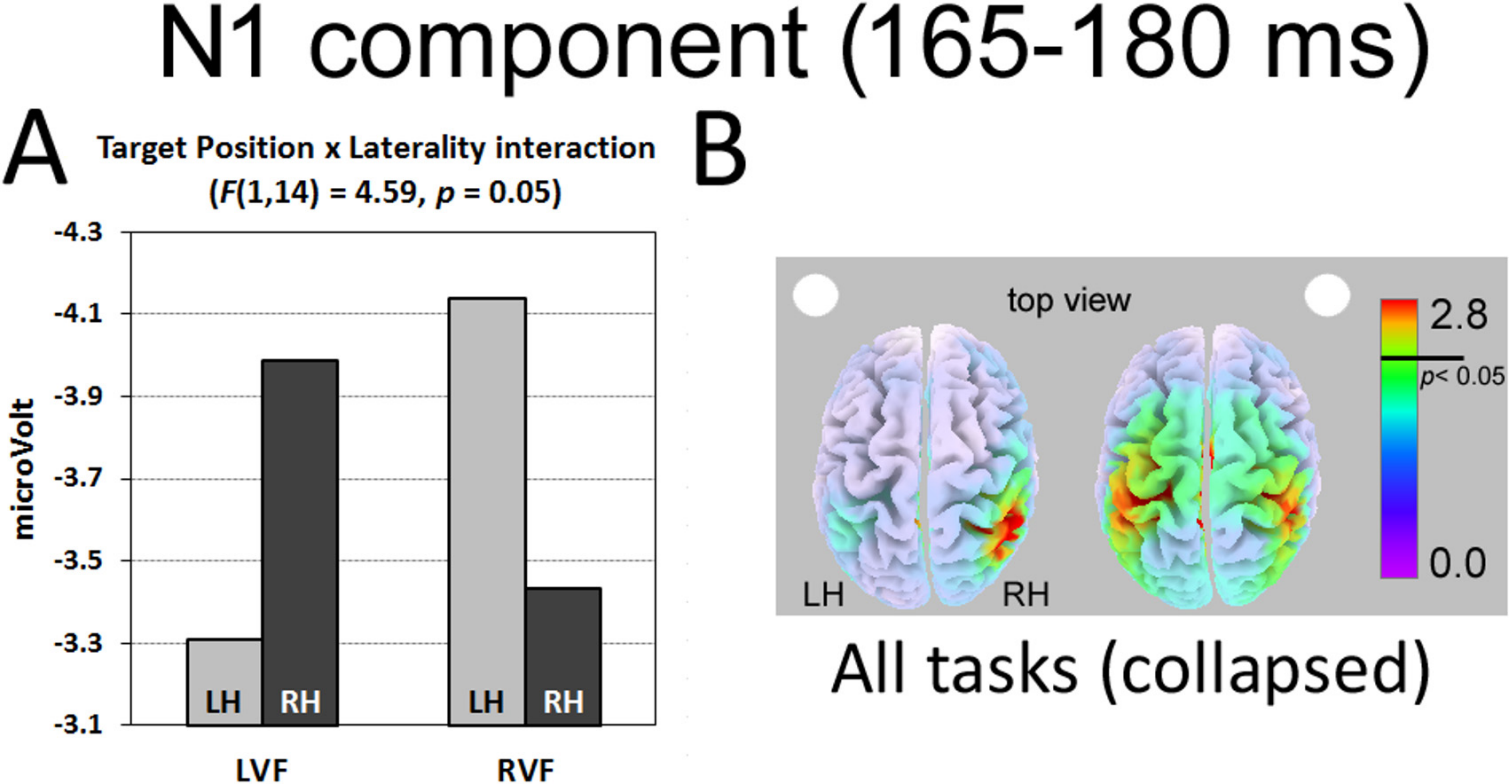
N1 component elicited on posterior sites. (A) Target position by Laterality interaction and (B) sLORETA source analyses of LVF and RVF stimuli collapsed across all tasks. The white dot indexes target position (left vs. right). LH = Left Hemisphere; RH = Right Hemisphere.

N1 component (posterior sites). The ANOVA carried out in the time interval corresponding to the N1 component (165–185 ms after target onset) showed a significant Target Position by Laterality interaction (F(1,14) = 4.59, p = 0.05), with increased amplitude for contralateral targets in the absence of any significant main effect (LVF target d = 0.14; RVF target d = 0.21; Fig 5A). No other main effect or interaction was significant.

**Fig 6:**
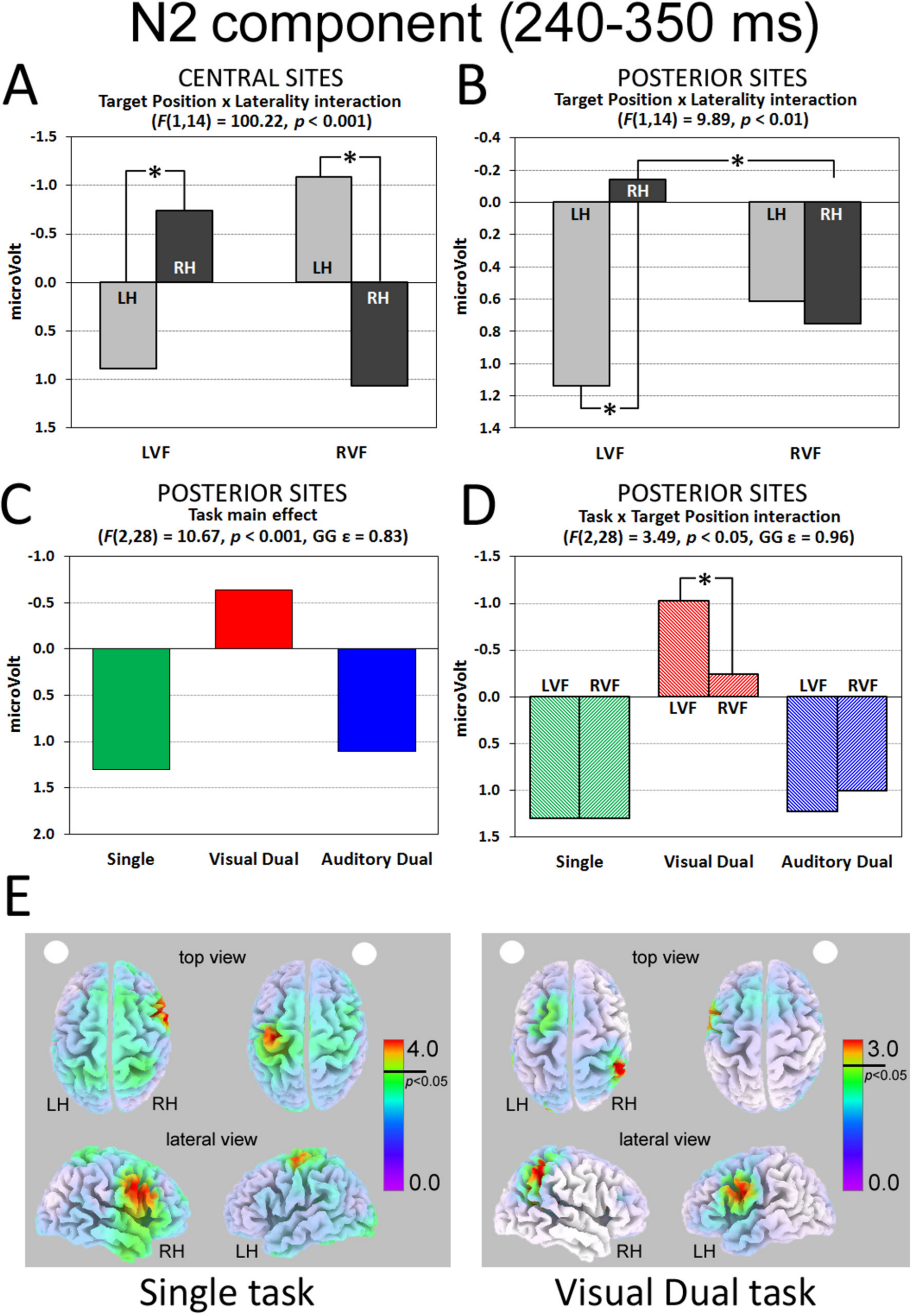
N2 component. Target Position by Laterality interaction on (A) central and (B) posterior sites. On posterior sites only, Task main effect (C) and Task by Target Position interaction (D) were significant. (E) sLORETA source analyses of LVF and RVF stimuli during Single (left panel) and Visual Dual tasks (right panel). The white dot indexes target position (left vs. right). * significant post-hoc comparisons. LH = Left Hemisphere; RH = Right Hemisphere.

N2 component (central sites). The ANOVA carried out in the time interval corresponding to the late N2 component (240–350 ms after target onset) showed a significant Target Position by Laterality interaction (F(1,14) = 100.22, p < 0.001): significant greater negativity was found contralateral to the side of target presentation, in a symmetric fashion (i.e., LVF targets elicited larger right than left negativity [p < 0.001, d = 1.05], whereas RVF targets evoked greater left than right negativity [p < 0.001, d = 1.42]; Fig 6A). No other main effect or interaction was significant.

N2 component (posterior sites). The ANOVA carried out in the time interval corresponding to the late N2 component (240–350 ms after target onset) showed a significant Target Position by Laterality interaction (F(1,14) = 9.89, p < 0.01). LVF targets elicited larger right vs. left negativity (p < 0.01, d = 0.49), whereas RVF targets evoked bilateral activation (d = 0.05, Fig 6B). With respect to within-hemisphere differences, greater right negativity was measured for RVF compared with LVF targets (p < 0.05, d = 0.32; Fig 6B).

In addition, a significant main effect of Task emerged (F(2,28) = 10.67, p < 0.001, GG ε = 0.83). In contrast to the absence of load effect for central sites, for posterior sites the Visual Dual task elicited greater negativity (-0.64 μV) than both the Single task and the Auditory Dual task (1.30, d = 0.77, and 1.11 μV, d = 0.78, respectively; all ps < 0.001; Fig 6C). Interestingly, the two-way interaction Task by Target Position was significant (F(2,28) = 3.49, p < 0.05, GG ε = 0.96), indexing different effects of the dual task manipulation depending on target position. Indeed, significant greater negativity was elicited by LVF vs. RVF targets (p < 0.01, d = 0.32) selectively in the Visual Dual task, whereas neither in the Single nor in the Auditory Dual task significant LVF/RVF differences were found, d = 0.01 and 0.11, respectively; Fig 6D). Furthermore, the Visual Dual task showed the greatest negativity for both target positions when compared with the other two tasks (all p < 0.001, LVF target d = 0.89 and d = 0.92; RVF d = 0.62 and 0.60). No other main effect or interaction was significant.

### Source analyses

The distributed source solution of every task-related component was computed by sLORETA/eLORETA separately for each condition. On the basis of ERP analyses, to localize the effect of load, we compared only significant sLORETA/eLORETA sources obtained in Single and Dual tasks collapsed (P1 component), all tasks collapsed (N1 component) and Single, Visual Dual, and Auditory Dual tasks (N2 component). We carried out separate t-tests by contrasting the P1, N1 and late N2 intervals (105–115, 165–185 and 240–350 ms after target onset, respectively) with an interval with no active visuo-perceptual processing (10-ms, 20-ms and 110-ms baseline prior to target onset, respectively). All results are reported in Talairach coordinates [50].

sLORETA/eLORETA analyses carried out on all participants revealed significant activity in the P1, N1 and late N2 intervals (all p < 0.01). Source analyses carried out separately on Single and Dual tasks collapsed located the cortical generator of the P1 component in the right and left Supramarginal Gyrus (Table 2 and Fig 4C). In addition, the prominent and significant occipital activation present during the Single task was not visible (and it was not statistically significant) under load (see Fig 4C, back view). The disappearance of occipital components under dual-tasking suggests that attentional load can already affect the first stages of visual processing and possibly transform target detection into a more effortful task.

**Table 2:** Source Analysis. The cortical sources found with sLORETA/eLORETA are reported in Talairach coordinates, together with other significantly active cortical areas separately for P1, N1 and P2 components and for target side.

**P1 component**	***Task***	**Structure name**	**BA**	**x**	**y**	**z**	**t**_**14**_
LVF	*Single*	right SMG	40	29	-29	21	3.82**
		right LG	18/17	15/20	-97/-92	-4/0	3.01/2.96**
		right MOG	18/19	30	-92	0/14	2.90/2.87**
		Cuneus	17	0	-97	1	2.76*
	*both Dual*	right SMG	40	35	-28	38	2.85**
		right IPL	40	40	-32	34	2.77**
RVF	*Single*	left SMG	40	-29	-39	21	3.80**
		left LG	18	-10	-97	-4	2.84**
		Cuneus	17	0	-97	1	2.79**
		left/right MOG	18	-25/25	-92	5/9	2.71/2.70**
		left MOG	19	-25	-87	9	2.69*
	*both Dual*	left SMG	40	-19	-33	37	3.70**
		left IPL	40	-35	-37	39	3.20**
**N1 component**	***Task***	**Structure name**	**BA**	**x**	**y**	**z**	**t**_**14**_
LVF	*All*	right SPL	40	36	-41	46	2.94**
RVF	*All*	left/right SMG	40	-19/20	-33/-30	37/40	3.47/3.26**
		left IPL	40	-35	-37	39	3.33**
**N2 component**	***Task***	**Structure name**	**BA**	**x**	**y**	**z**	**t**_**14**_
LVF	*Single*	right PrG	6	58	5	35	3.98**
	*Visual Dual*	right SMG	40	40	-35	33	2.84**
	*Auditory Dual*	right PrG	6	49	-3	38	2.65**
RVF	*Single*	left PrG	6	-35	-11	59	3.87**
	*Visual Dual*	left PrG	6	-58	0	29	2.96**
	*Auditory Dual*	left IFGtr/PrG	44	-53	17	11	2.84**

** *p* < 0.01

* *p* < 0.05, two-tailed.

BA = Brodmann area; SMG = supramarginal gyrus; LG = lingual gyrus; MOG = middle occipital gyrus; IPL = inferior parietal lobule; SPL = superior parietal lobule; PrG = precentral gyrus; IFGtr = inferior frontal gyrus–triangular part.

The cortical generators of N1 component elicited during all tasks collapsed were located in right Superior Parietal Lobule (approximate coordinates for LVF targets: 36, -41, 46) and left and right Supramarginal Gyri as well as in left Inferior Parietal Lobule (approximate coordinates for RVF targets: -19/20, -33/-27, 37/38 and -35, -37, 39; Table 2 and Fig 5B).

Interestingly, the cortical generators of the late N2 component were located in different portions of right/left Precentral Gyrus, depending on the position of the targets, for all tasks (see Table 2 for approximate coordinates). The only exception was the Visual Dual task, which mainly activated the right Supramarginal Gyrus when LVF targets were presented (approximate coordinates: 41, -35, 59; Fig 6E).

### Cost analyses

Finally, to focus specifically on load effects, we carried out an additional ANOVA on P1, N1 and N2 components of Dual task minus Single task activity. This allowed us to directly compare the costs of intra-modal load (Visual Dual task minus Single task) and cross-modal load (Auditory Dual task minus Single task). Thus, separate ANOVAs on P1, N1 and N2 time-intervals were performed including Task (two levels: Visual Dual vs. Auditory Dual), Target Position (two levels: LVF vs. RVF) and Laterality (two levels: Left vs. Right hemisphere) as factors.

**Fig 7:**
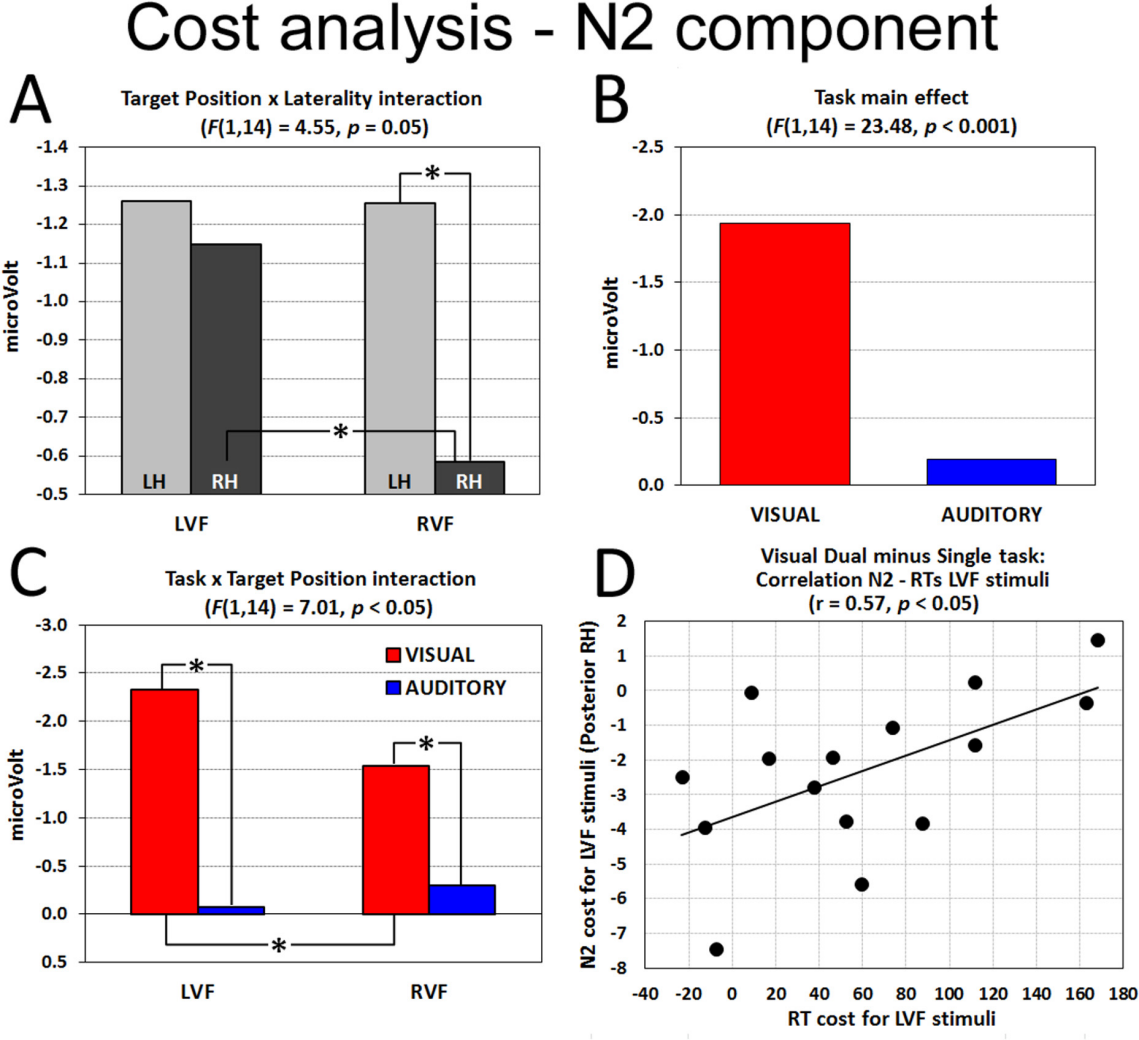
Cost analysis. (A) Stimulus Position by Laterality interaction, (B) Task main effect and (C) Task by Target Position interaction. (D) Correlation between behavioral (RTs) and electrophysiological costs for LVF targets in the Visual Dual task: the greater the increase of activation in posterior right sites, the faster the RTs. * significant post-hoc comparisons. LH = Left Hemisphere; RH = Right Hemisphere.

No significant effect was found in the analysis of the P1 component, confirming the previous analyses and suggesting that both visual and auditory dual tasks elicited greater positivity than the single task. With respect to N1 component, notwithstanding that the Task by Target Position interaction was significant (F(1,14) = 4.70, p < 0.05), post-hoc comparisons showed distributed effects, since no differences between tasks and target position reached significance. Also the analysis of the late N2 component revealed no effects on central sites. In contrast, cost analysis on posterior regions showed a significant Target Position by Laterality interaction (F(1,14) = 4.55, p = 0.05). Regardless of task, LVF targets elicited the same cost levels on both left and right posterior sites (d = 0.04), whereas RVF targets mainly activated left rather than right cluster (p < 0.01, d = 0.32; Fig 7A). With respect to within-hemisphere differences, greater right negativity was measured for LVF compared with RVF targets (p < 0.01, d = 0.24), suggesting higher costs for targets presented in the LVF under high load.

On posterior sites, however, regardless of the laterality distribution of N2 component, the effect of load was significantly different between intra-modal and cross-modal conditions (Task main effect: F(1,14) = 23.48, p < 0.001): the Visual Dual task elicited greater costs than the Auditory Dual task (d = 0.82; Fig 7B). In addition, the Task by Target Position interaction (F(1,14) = 7.01, p < 0.05) revealed greater costs during intra-modal load (Visual Dual task) with respect to cross-modal load (Auditory Dual task) for both LVF and RVF (all ps < 0.001, d = 0.98, and 0.63, respectively; Fig 7C). Furthermore, within the Visual Dual task, LVF targets elicited greater costs than RVF ones (p < 0.01, d = 0.41). No other main effect or interaction was significant.

To determine whether the increased N2 activation for the Visual Dual task, and particularly for LVF targets, was functional or dysfunctional for task execution, we carried out a Pearson’s correlation between electrophysiological and behavioral cost measures. Participants’ RT cost for LVF targets, obtained by subtracting mean RTs in the Single task from mean RTs in the Visual Dual task, was correlated with the individual N2 cost over right posterior sites. As can be seen in Fig 7D, the smaller the RT cost in the behavioral responses to LVF targets, the larger the N2 cost (r(13) = 0.57, p < 0.05), suggesting that the increased activation (load cost) is functional to a more efficient processing of LVF targets under intra-modal load.

## Discussion

The present study focused on the electrophysiological correlates of those situations where the peripheral space has to be continuously monitored, while participants allocate additional attentional resources either in the visuo-spatial or in the auditory domain. This condition mimics, in a controlled environment, a number of everyday life contexts whereby spatial processing is performed under multitasking. In this condition spatial attention is not only deployed in the foveal space to efficiently process visual items, but it has also to continuously monitor the surrounding space, while being also flexible enough to effectively process and respond to information from non-visual sensory channels. The electrophysiological correlates of multitasking were investigated by comparing a condition where attention was only engaged to monitor space for the appearance of relevant items, with other conditions where attention was also deployed towards different stimuli and sensory channels to process, in parallel, either visual or auditory information.

Participants visually monitored the surrounding visual space for the appearance of briefly-presented lateralized targets, which were spatially categorized as left-sided or right-sided (all tasks). In this regard, it is worth noting that the eccentricity of the spatial positions to be monitored was relatively large, as opposed to the standard RSVP paradigm [51]. In addition, for Dual tasks only, participants had to pay attention and to verbally classify either a centrally-presented shape (Visual Dual task) or a binaurally presented sound (Auditory Dual task). It is important to point out that, together with the lateralized dot, the other two types of stimuli (i.e., the central shape and the sound) were always presented in all conditions, even when they were not task-relevant. This manipulation of task instructions allowed us to obtain a pure measure of the top-down attentional load induced by dual-tasking, without changes of the sensory information available to the participants. The cross-modal load condition (i.e., Auditory Dual task) included in our paradigm allows to disentangle visual load from a general effect on unspecific task demands. Moreover, this condition is both non-spatial and non-visual, whereas other studies have adopted as non-spatial conditions non-lateralized visual tasks that do not allow to explore the effects of cross-modal load [36].

Participants’ RTs confirmed the effect of increased task demands, with slower responses to targets in both Dual tasks in comparison to the Single task condition. Electrophysiological data provided a wealth of additional information. The analysis of the first positive component (P1), which is a marker of the allocation of spatial attention [52], revealed distinct patterns of posterior activation as a function of target position. [It could be argued that our paradigm does not allow to disentangle the ERP response to central vs. peripheral stimuli. Nevertheless, on the basis of P1 component results, we are confident that the ERPs genuinely reflect the processing of peripheral (target) stimuli. Indeed, regardless of load effects, the P1 component revealed significant right lateralization for LVF stimuli and bilateral activation for RVF stimuli: if ERP modulations were only the results of central stimulus processing, there would have been no sign of laterality patterns]. That is, RVF targets elicited a similar activation across hemispheres, whereas LVF targets showed reduced amplitude within the left and increased amplitude within the right hemisphere. This pattern of activation is in agreement with the hypothesis that spatial attentional orienting is a result of the interplay between attentional vectors and hemispheric hyper/hypoactivity [53]. Indeed, according to Kinsbourne’s proposal, spatial attention orienting results from the interaction of two antagonistic vectors, one for each hemisphere. Each vector implements spatial attentional orienting by means of a gradient that is characterized by minimal processing of ipsilesional spatial information and maximal processing of contralesional spatial information. After brain damage, the affected hemisphere is supposed to be no longer able to effectively contrast the contralateral orienting effect exerted by the unaffected hemisphere. Strong empirical evidence for this explanation arises from the beneficial effects (i.e., improvements in contralesional spatial awareness) of inhibitory TMS over the left frontal cortex of the contralesional hemisphere [54]. To explain the higher incidence of neglect after right hemisphere lesions the model assumes that–already in intact cognitive architectures–the two vectors are not equivalent, given that the right hemisphere creates a balanced gradient whereas the left hemisphere would be more effective in processing contralesional items.

With regard to the effect of multi-tasking, a key finding of the present study is that the effect of load was already visible about 110 ms after target onset, as indexed by increased P1 activation in context of higher processing demands (Dual tasks) regardless of the nature of the concurrent task. The effect of load on a component (i.e., the P1) known to be sensitive to the automatic allocation of spatial attention is of paramount importance because it might represent the first evidence of multimodal, top-down attention effects upon task-relevant targets. This finding suggests that our multitasking manipulations successfully modulated attention at the earliest phases of stimulus processing. It is worth noting that the administration of tasks in a partially fixed sequence, with the Single task always performed before the Dual tasks, played against the increase of P1 amplitude observed in the dual task conditions because repeated stimuli usually elicit a decrease in P1/N1 amplitude (repetition suppression [55]).

The source analysis located the main generators of P1 component in right and left SMG (for LVF and RVF targets, respectively), in agreement with neuroimaging studies that highlighted a key role of TPJ in the (re)orienting of spatial attention [56,57] and in mediating the imbalance between hemispheres [58]. In addition, the prominent occipital activation that was present during the Single task (Table 2 and Fig 4C, back view) disappeared during both dual tasks, in which only the Supramarginal Gyri and the Inferior Parietal Lobules were active. Thus, in the Single task, the activation of Supramarginal gyri was associated with that of the extrastriate cortices, indexing a distributed parieto-occipital pattern of cortical activation. In contrast, during both dual tasks, the activation was mainly limited to the Supramarginal gyri, suggesting a suppression of activity in the early, occipital, visual areas, in accordance to what have been suggested by fMRI studies in healthy participants as well as in right brain damaged patients [26, 13]. The detrimental effect of load on primary sensory areas observed with the high temporal resolution characterizing ERPs allows to rule out the possibility that previous fMRI findings were only due to absence of feedback activation, which would have been erroneously considered as an early modulation due to the poor temporal resolution of the technique.

While multitasking affected the earliest positive ERP component, its effect was not evident in the subsequent (but still rather early) component, the N1 (165–180 ms after stimulus onset), which showed the standard enhancement for contralateral with respect to ipsilateral targets [39]. Indeed, significant lateralized N1 negativity–contralateral to the side of target onset–was present regardless of task condition. The source analysis located the main generators of N1 component in the right Superior Parietal Lobule (LVF targets) and in the left/right Supramarginal Gyri and left inferior Parietal Lobule (RVF targets), in agreement with functional neuroimaging evidence [59–61].

The combined effects of target position and attentional load showed up in the late N2 component. Inspecting the grand-mean waveforms (Figs 2 & 3) over the central sites, it can be noticed that this later time interval corresponds to the preparation of motor response (see the greater negativity/activation on right vs. left electrodes for LVF targets, and the reversed pattern for RVF targets). At the same time, however, a concurrent negativity/activation appeared on posterior sites. The statistical analysis carried out on this temporal window confirmed these patterns: central sites showed greater activation for contralateral targets, regardless of the type of task. At posterior sites, the asymmetric pattern of activation–already evident in P1 and N1 components (i.e., increased contralateral negativity/activation for LVF targets and bilateral activation for RVF targets) continued at this later stage. Most notably, the Visual Dual task differed from both Single and Auditory Dual tasks, showing much greater activation as well as a marked asymmetry in favor of LVF targets. Source analyses carried out on the late N2 component located the cortical generator of this particular condition (i.e., LVF target processing in the Visual Dual task) in the right SMG. The cortical sources of all other conditions were, instead, located in different portions of the PrG (Table 2). These results suggest that the effect of the visual load could be well appreciated on the right hemisphere, with increased activation at posterior sites. In particular, the late activation of the right SMG could reflect the effort to voluntarily allocate attention under visual load conditions. Accordingly, it is conceivable that the significant correlation between increased N2 negativity/activation in right posterior sites and smaller RT costs to LVF targets indexes the recruitment of neural resources that are functional for a more efficient processing of the left hemispace under intra-modal load. This was not the case for the cross-modal load condition, which implies attentional allocation to different sensory channels, as attested by the lack of significant differences between Single task and Auditory Dual task. Crucially, the latter finding does not stem from a different level of difficulty or performance in the two types of dual task. Indeed, participants’ RTs and accuracy rates did not differ between Visual and Auditory Dual tasks. It is also worth noting that the distinct electrophysiological patterns elicited by Visual and Auditory Dual tasks rule out the hypothesis that the effect of multi-tasking is aspecific and the possibility that it might simply reflect the introduction of a second response as part of the task demands.

The comparison of our investigation with the only previous ERP study that to our knowledge employed peripheral, task relevant targets in the presence of variable attentional load at fixation [41] reveals both commonalities and differences. Both studies found increased (central) P1 following higher load at fixation. However, whereas the P1 modulation we found was obtained under dual tasking (spatial monitoring + concurrent task) in [41] it occurred as a consequence of increased load at fixation, in the absence of peripheral targets. In [41] the effects of visual load extended to N1. However, any comparison should be cautious due to a number of methodological differences in the spatial task to be performed (detection in their study vs. localization in ours) as well as in the load manipulations (in their study: absence of an auditory load condition, central stimulus never presented concurrently with targets, central task requiring a yes/no response).

The increased P1 indexes that attentional orienting is more difficult under conditions of high load, regardless of the sensory modality (i.e., intra- vs. cross-modal) of the concurrent task, and requires, from very early phases of target processing, the recruitment of (right hemisphere) posterior areas devoted to spatial attention. Indeed, fMRI evidence suggests that the right hemisphere is crucial for maintaining attention to spatial locations over time [62]. In particular, the right TPJ has been conceived as a “circuit breaker” for the dorsal system [63]. The “reorienting” response would result from the coordinated action of a right-hemisphere ventral fronto-parietal network, that interrupts and resets ongoing activity, and a dorsal fronto-parietal network, specialized for selecting and linking stimuli and responses. Therefore, our data might be compatible with neuroimaging evidence of ventral network suppression to prevent reorienting to distracting events under condition of attentional engagement [64], a mechanism that is thought to be evolutionarily selected for preventing sensory overload. Source analyses showed that, also at later stages (i.e., N2 component) left- and right-sided stimuli are processed differently, at least in the high-demanding condition of our experimental paradigm. Indeed, during visual Dual task, right-sided targets elicited a contralateral activation of premotor areas, whereas left-sided targets mainly activated the right SMG. The late recruitment of right-hemisphere areas might be necessary for participants to comply with a high-demanding task requiring the processing of multiple information sources within the visual modality. In contrast, targets presented in the right hemifield recruited more consistently areas related to automatic, effortless processing, such as the left Frontal Eye Fields (FEF). Also this finding is compatible with TPJ suppression occurring at early stages of concurrent task encoding [22] and then resolving at later stages. TMS studies which focused on the role of contralateral posterior cortex in visual perception confirm the key top-down modulation deriving from parietal cortex described in brain-damaged patients with fMRI [13]. Visual cortical excitability is increased by TMS over unilateral posterior parietal cortex (PPC) but it can be abolished by bilateral PPC stimulation [65]. Crucially, in right-hemisphere-damaged patients, extinction at double stimulation can be reduced when contralesional TMS is applied [52]. Both findings confirm that the two hemispheres compete for attentional, top-down, modulation upon spatial processing and awareness, at the top of the functional asymmetry (left hemisphere mainly controlling contralateral space) highlighted by early components independently of task load. The influential model of Corbetta and Schulman [7] suggests that the core deficits leading to neglect are not uniquely spatial, as often assumed, but also non-spatial. The dual-tasking approach we have implemented might, in the future, also be helpful for better determining the degree of overlap between attention and working memory-related processes [66].

In summary, the present study focused on the ERP correlates of increased task demands upon spatial monitoring, using a paradigm that, in right-hemisphere-damaged patients, was shown to induce a severe disruption of spatial awareness for contralesional hemispace [15]. Though the behavioral performance of the healthy participants was affected by concurrent task performance only in terms of slower responses to the peripheral targets, without any interaction with target side, ERP revealed differences between hemispaces as well as between within- and cross-modal load. Modality-independent effects were found for the P1 component, where both visual and auditory load modulated this early component and suppressed the signal in the early visual areas. Modality-specific effects were found for the N2 component, and were more marked in the right hemisphere. These results suggest that the right hemisphere is particularly affected by load manipulations, in agreement with its crucial role in subtending automatic orienting of spatial attention. The load-dependent (and modality-independent) modulation of early components might be the substrate on which deficits for contralesional hemispace emerge under heterogeneous, attention-demanding conditions in right hemisphere-damaged patients.
